# Accuracy of a Semi-Quantitative Ultrasound Method to Determine Liver Fat Infiltration in Early Adulthood

**DOI:** 10.3390/diagnostics10060431

**Published:** 2020-06-25

**Authors:** Camila Ibacahe, Paulina Correa-Burrows, Raquel Burrows, Gladys Barrera, Elissa Kim, Sandra Hirsch, Boris Jofré, Estela Blanco, Sheila Gahagan, Daniel Bunout

**Affiliations:** 1 Instituto de Nutrición y Tecnología de los Alimentos, Universidad de Chile, Santiago de Chile 783490, Chile; camila.ibacache.alvarez@gmail.com (C.I.); rburrows@inta.uchile.cl (R.B.); gbarrera@inta.uchile.cl (G.B.); shirsch@inta.uchile.cl (S.H.); dbunout@inta.uchile.cl (D.B.); 2 Division of Child Development and Community Health, University of California San Diego, La Jolla, CA 92093, USA; elissakim3@gmail.com (E.K.); esblanco@health.ucsd.edu (E.B.); sgahagan@health.ucsd.edu (S.G.); 3 Servicio de Gastroenterología, Clínica Santa María, Santiago de Chile 7520378, Chile; 4 Servicio de Radiología e Imagenología, Clínica Santa María, Santiago de Chile 7520378, Chile; bjofrea@yahoo.es

**Keywords:** fatty liver, ultrasound, validity assessment, cardiometabolic risk, obesity

## Abstract

An inexpensive and simple method to determine non-alcoholic fatty liver disease (NAFLD) is the abdominal ultrasound, but there are still doubts about its accuracy. We assessed the precision of a semi-quantitative ultrasound method to determine liver fat infiltration, using magnetic resonance spectroscopy (MRS) as the reference. The study was conducted in youths from an ongoing cohort study. Clinical validation was performed, using receiver operating characteristic analysis, in *n* = 60 participants (22.6y; 50% males). Abdominal ultrasound was carried out with liver brightness (score 0–3), diaphragm attenuation (0–2) and liver vessel blurring (0–1) scored by two observers. Liver fat was estimated using MRS. Then, analytical validation was conducted in the remaining participants (*n* = 555; 22.7y; 51% males) using effects size estimates. An ultrasound score ≥4.0 had the highest sensitivity (78%) and specificity (85%) for NAFLD diagnosis. An area under the curve of 86% denotes a good diagnostic performance of the test, whereas a Kappa of 0.63 suggests substantial agreement of ultrasound vs. MRS. The analytical validation showed that participants having NAFLD according to ultrasound had an unhealthier cardiometabolic profile than participants without the condition. Abdominal ultrasound, combined with a semi-quantitative score system, is a reliable method to determine liver fat infiltration in young adults and should be encouraged whenever MRS is unavailable.

## 1. Introduction

Excessive liver fat accumulation not associated with alcohol abuse, or non-alcoholic fatty liver disease (NAFLD), may progress to steatohepatitis, cirrhosis and hepatoma. It is closely associated with increasing obesity prevalence among adults; thus, it is rapidly becoming the most common liver disease worldwide [1]. NAFLD is highly prevalent in all continents, but the highest rates are reported in the Middle East (32%), South America (31%), Asia (27%), the United States (24%) and Europe (23%) [2]. In Chile, a study conducted with ultrasound showed that 23% of participants had NAFLD [3], slightly below the global prevalence rate [3]. The same study also found that a significant risk factor was a body-mass index (BMI) over 26.9 kg/m^2^ [3]. Currently, 74.2% of the Chilean population >15 years have some degree of excess weight [4]. Since obesity is a public health issue nationally and internationally and because individuals with NAFLD are at high risk of developing cardiometabolic comorbidities that could place growing pressure on health-care systems [5], an easy low-cost diagnostic method for NAFLD is needed.

The large number of individuals with or at risk of NAFLD and the rising incidence among younger age populations [2,6] create challenges for screening. Furthermore, clinical guidelines for obesity treatment and prevention recommend early detection of liver function before more serious complications result [7,8]. Non-invasive assessment of liver fat infiltration may be carried out using imaging studies. Among these, ultrasound is the most widely used method in clinical settings, but there are doubts about its accuracy for estimating the amount of liver fat [9,10] adequately. Ultrasound is non-invasive, inexpensive and does not expose patients to radiation. To improve its accuracy, Hamaguchi et al. proposed a scoring system based on the comparison of liver and kidney echogenicity, assessment of liver brightness, of deep attenuation, of diaphragmatic contours by the liver, and of liver vessel blurring. They reported an almost perfect within- and between-observer agreement, and very good sensitivity and specificity of the scoring system for diagnosing NAFLD in patients who had undergone liver biopsy [11]. This scoring system reduces the inter-observer variation, considering that ultrasound is an operator-dependent imaging technique [12]. The accuracy of computed tomography for the diagnosis of NAFLD is approximately the same as ultrasound [13].

Magnetic Resonance Spectroscopy (MRS), on the contrary, is an accurate method to quantify liver fat, when compared with liver biopsies [14]. It has the advantage of not exposing patients to ionizing radiation but is expensive, not universally accessible, and not available for preventive purposes. Due to its accuracy, it can be used as the gold standard to compare other techniques to detect NAFLD, as reported previously [15].

We aimed to assess the accuracy of abdominal ultrasound to diagnose NAFLD in male and female young adults across all ranges of nutritional status. Assessment of liver fat infiltration was performed with the score proposed by Hamaguchi et al. and MRS was used as the reference method for comparison. Clinical validity or the ability of the test to detect the presence or absence of NAFLD was complemented with assessments of its analytical validity, which defines the capacity of the test to measure accurately and reliably a phenotype of interest.

## 2. Materials and Methods

### 2.1. Participants and Study Design

A cross-sectional study was conducted in 22–23 year-olds from the Santiago Longitudinal Study, who were recruited as infants from low-to-middle income neighborhoods in Santiago, Chile. Growth and developmental assessments were performed at multiple time-points from enrollment (4 months) through adulthood [16,17,18]. At 22–23y, they were assessed for the presence of obesity and cardiometabolic risk [18]. Of *n* = 630 participants, *n* = 15 (2%) had suboptimal ultrasound images and, thus, were excluded from the study. A total of *n* = 60 were included in the clinical validity assessment; they had no history of alcohol or illegal drug abuse and normal renal function. The remaining participants (*n* = 555) were involved in the analytical validation. Ethical approval was obtained by the institutional review boards of the University of Michigan, Institute of Nutrition and Food Technology (University of Chile), and the University of California, San Diego. Informed and written consent was provided according to the norms for Human Experimentation, Code of Ethics of the World Medical Association (Declaration of Helsinki, 1995).

### 2.2. Measurements

Participants were assessed on a single day in the fasting state. They all underwent anthropometric examination, cardiometabolic assessment and abdominal ultrasound. Those enrolled in the clinical validity also had MRS of the liver.

#### 2.2.1. Anthropometric Assessments

Research personnel performed the anthropometric examination. Standardized procedures were used to measure the height (cm) to the nearest 0.1 cm, using a Holtain stadiometer, and weight (kg) to the nearest 0.1 kg, using a scale (Seca 703, Seca GmbH & co. Hamburg, Germany). Waist circumference was measured with a non-elastic flexible tape and recorded to 0.1 cm (Seca 201, Seca GmbH & co. Hamburg, Germany). Measurements were taken twice, with a third measurement if the difference between the first two exceeded 0.3 kg for weight, 0.5 cm for height and 1.0 cm for WC. BMI was calculated and nutritional status was evaluated with the World Health Organization references.

#### 2.2.2. Cardiometabolic Assessment

After the anthropometric assessment and 15 min rest, systolic and diastolic blood pressures were measured three times, according to the US Preventive Services Task Force Recommendation Statement (2015) using a standard mercury sphygmomanometer. A blood sample was obtained to measure blood glucose, insulin, blood lipid levels, adiponectin, high-sensitivity C-reactive protein, total bilirubin, alkaline phosphatases, alanine and aspartate aminotransferase, gamma-glutamyl transferase, hepatitis B surface antigen and hepatitis C virus antibodies. The homeostatic model assessment to quantify insulin resistance (HOMA-IR) was estimated (HOMA-IR = [insulin(µU/l) × glucose(mg/dl) × 0.0555]/22.5]) and values ≥2.6 were considered insulin resistance [19]. Metabolic Syndrome was diagnosed based on the 2009 International Diabetes Federation/American Heart Association/National Heart, Lung and Blood Institute Joint Interim Statement [20].

#### 2.2.3. Abdominal Ultrasound

**Figure 1:**
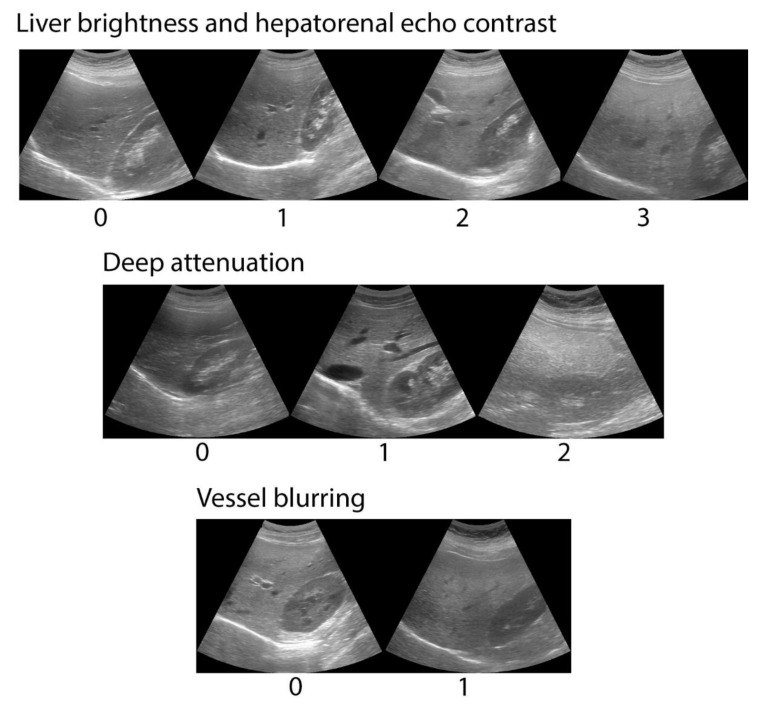
Examples of abdominal ultrasound images combined with the semi-quantitative score system by Hamaguchi et al. to assess liver fat infiltration in Chilean young adults. The system scores liver brightness from 0 to 3, diaphragm attenuation from 0 to 2 and vessel blurring from 0 to 1. Maximum score possible was 6.

An abdominal ultrasound was performed using a General Electric LogiQ ultra-sonographer with a 4C RS convex multifrequency probe (2–5.5 MHz) (GE Healthcare Systems, Wauwatosa, WI, USA). All examinations were done by the same operator, who obtained and stored the images that were analyzed by two independent observers. The operator was trained by experienced ultra-sonographers to obtain standardized images in which the liver and the right kidney would be seen simultaneously. Images were obtained with the participant rolled onto their left side in a decubitus position, with their right arm stretched above the head after taking a deep breath. Observers were gastroenterologists with training in abdominal ultrasound interpretation, and scored liver brightness from 0 to 3, diaphragm attenuation from 0 to 2 and vessel blurring from 0 to 1, according to Hamaguchi [11]. Examples of ultrasound images are shown in Figure 1. The maximum score possible was six. When there was disagreement between observers, images were analyzed jointly and agreement about the final score was reached.

#### 2.2.4. Magnetic Resonance Spectroscopy

On the same day, an MRS of the liver was done at a clinical facility using a Siemens Magnetom 1.5T resonator (Magnetom Symphony, Siemens Medical, Erlangen, Germany). Images were obtained with the participant in supine decubitus and in apnea. A modification of the Dixon method involving a multi-breath-hold double gradient-echo T1-weighted sequence was used [15,21]. In-phase and out-of-phase images were obtained, and a region of interest with the highest amount of fat was identified. A comparison of the amount of fat in the liver and in the region of interest was calculated [21]. LiverLab software from Siemens was used to quantify liver fat. NAFLD was diagnosed when liver fat exceeded 5%, according to the NASH Clinical Research Network Scoring System [22]. An expert radiologist reviewed all the images to confirm the results.

### 2.3. Data Analysis

All analyses were carried out using Stata 15 for Windows (Lakeway Drive College Station, Texas, USA). Normality of distribution was determined using the Shapiro Wilk test, and variables were described accordingly. Student’s *t* test, Wilcoxon’s rank-sum test, and χ^2^ test were used to compare groups.

#### 2.3.1. Clinical Validity

Receiver operating characteristic analysis was used to find the optimal cut-off of Hamaguchi score for echogenicity for NAFLD diagnosis. Sensitivity, specificity, the area under the curve, likelihood ratio and post-test probability were estimated. The Youden Index [sensitivity-(1-specificity)] was calculated to determine the optimal cut-off for NAFLD diagnosis. Additionally, Cohen’s Kappa (κ) coefficient was calculated to assess the agreement between ultrasound and MRS for the diagnosis of NAFLD. Cohen’s Kappa was estimated as follows:

κ = (p_o_ − p_e_)/(1 − p_e_) = 1 − (1 − p_o_)/(1 − p_e_) (1)

where p_o_ is the relative observed agreement among tests and p_e_ is the hypothetical probability of chance agreement. Kappa was interpreted using Landis and Koch qualitative scale [23]. Ultrasound scores of the two observers were compared before reaching an agreement. The correlation between the difference and the mean of both scores was also calculated. The significance of this correlation was calculated using the Bradley-Blackwood equation [24]. A significant correlation or a significant slope of the curve indicates a lack of concordance. Kappa was also calculated to measure inter-observer agreement for the ultrasound diagnosis of NAFLD.

#### 2.3.2. Analytical Validity

We checked whether our ultrasound cut-off for NAFLD diagnosis was related to higher biological risk in the group having values ≥4. Cohen’s *d* and Cliff’s *δ* were used to indicate the standardized difference between mean and median values, respectively, of selected cardiometabolic biomarkers after controlling for the presence of NAFLD as predicted by the abdominal ultrasound. Values of *d* = 0.20, 0.50 and 0.80 denote small, medium and large differences between means [25], whereas, the absolute value of *δ* is considered small around 0.15, medium around 0.33, and large around 0.47 [26].

## 3. Results

### 3.1. Clinical Validity

We studied *n* = 60 participants aged 22.6 (22.3–22.7) years (50% women); their median BMI was 27.7 (23.3–32.5) kg/m^2^ and 37% had a BMI over 30 kg/m^2^ (Table 1). All had negative hepatitis B surface antigen and hepatitis C virus antibodies. According to MRS, 27 participants (45%) had hepatic fatty infiltration of ≥5%. Of these, 16 (59%) were males, and 18 (67%) had a BMI of ≥30 kg/m^2^.

**Figure 2:**
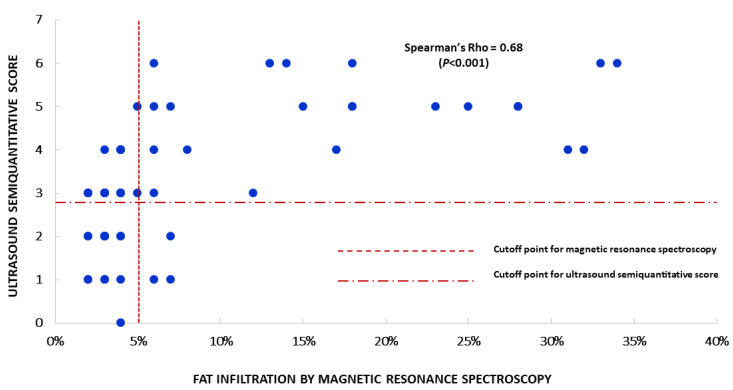
Plot of the ultrasound semi-quantitative score of liver fat versus the percentage of fat infiltration determined by magnetic resonance spectroscopy, in all participants. Each point denotes the score assigned to that given percentage of fat infiltration.

In the sample, the optimal cut-off of ultrasound score for NAFLD diagnosis was 4.0. At this point, the sensitivity and specificity of the method as a diagnostic tool were 78% and 85% (Table 2). After controlling for sex, sensitivity was 75% in males and 82% in females, whereas specificity was 86% in the former and 84% in the latter. An area under curve of 86% overall in the sample, 85% in males and 90% in females denotes a good performance of an ultrasound score of ≥4.0 to predict the presence of fat infiltration of ≥5%. Twenty-six participants (43%) had an ultrasound score of ≥4.0. Of these, 14 (54%) were males, and 16 (61%) had a BMI of ≥30 kg/m^2^. To further illustrate the concordance of ultrasound with MRS, the plot of the ultrasound score against the percentage of fat infiltration is depicted in Figure 2.

The ability to differentiate between pre- and post-test probabilities of a disease is a major factor in the indication of medical tests. The pre-test probability is given by the prevalence of the condition. In our participants, the pre-test probability of having NAFLD was 45% before the ultrasound. Afterwards, for those having an ultrasound score of ≥4, the chances of having the disease increased to 81%. Conversely, in participants having an ultrasound score below the optimal cut-off, the chances of having NAFLD reduced to 18% (Table 2).

Before reaching an agreement for the discordant scores, the Rho of the concordance between the two observers for the ultrasound score was 0.656 (*p* < 0.01). The Bradley-Blackwood F was 2.191 (*p* = 0.11). The Kappa concordance for the diagnosis of NAFLD was 0.47 (95% CI, 0.31–0.62), *p* < 0.01). In 13 participants, there was a disagreement between the two ultrasound raters for the diagnosis of NAFLD. Likewise, Kappa between fat infiltration of 5% or more by MRS and an ultrasound score of four or more was 0.63 (95% CI 0.37–0.88), denoting a substantial agreement. Kappa between each rater and MRS was 0.40 (95% CI 0.27–0.53) and 0.39 (95% CI 0.26–0.52) respectively, indicating a moderate agreement, according to Landis and Koch.

Table 3 shows the anthropometric and laboratory features of participants, separated by the presence of NAFLD according to ultrasound. As expected, those with NAFLD had a higher BMI. Also, they had higher blood pressure, insulin, and HOMA-IR, and a trend towards higher serum triglycerides. After controlling for sex, we observed that males with NAFLD had an unhealthier cardiometabolic profile compared to females in the same condition.

### 3.2. Analytical Validity

**Figure 3:**
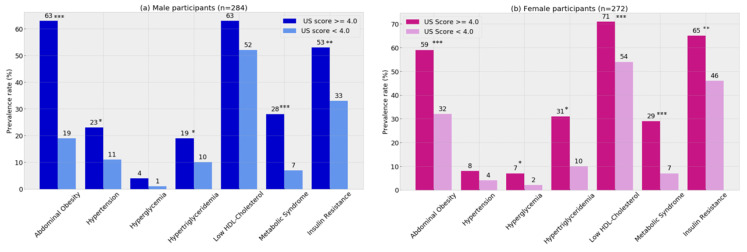
Cardiometabolic risk at 23y in the Santiago Longitudinal Study by NAFLD diagnosis with abdominal ultrasound: male (**a**) and female (**b**) participants Metabolic Syndrome and its components were diagnosed with the AHA/NHLBI/IDF joint standard. Insulin resistance was diagnosed with HOMA-IR values ≥ 2.6 in both male and female participants. Chi2 test for independence: * *p* < 0.05, ** *p* < 0.01, *** *p* < 0.001.

We studied *n* = 555 participants aged 22.7 (22.3–22.9) years (51% males); their median BMI was 25.6 (22.5–29.6) kg/m^2^ and 25% had a BMI over 30 kg/m^2^. When compared to participants enrolled in the clinical validity, we observed that those included in the analytical validity had lower BMI, systolic blood pressure, insulin and HOMA-IR. Hepatic transaminases were also lower in this group. Higher prevalence of abdominal obesity, hypertriglyceridemia, Metabolic Syndrome and insulin resistance were found in males and females having an ultrasound score of 4 compared to those having a score below the optimal cut-off for NAFLD diagnosis (Figure 3). Males with NAFLD according to ultrasound had also a higher prevalence of hypertension, whereas, females with NAFLD had a higher prevalence of low high-density lipoprotein cholesterol and hyperglycemia compared to participants not having NAFLD as predicted by the ultrasound. 

Table 4 contains the cardiometabolic profile of participants after controlling for sex and the presence of NAFLD according to ultrasound. Males and females meeting criteria for NAFLD according to ultrasound had significantly higher values of waist circumference, blood pressure, glycemia, insulin, HOMA-IR and adiponectin compared to participants without the condition. Males with NAFLD had increased high-sensitivity C-reactive protein (mg/l), an inflammatory biomarker, compared to males without NAFLD. Notably, in both sexes, the effect size for the difference was large for waist circumference and moderate for diastolic blood pressure and insulin.

## 4. Discussion

### 4.1. Main Findings

Ultrasonography had fair-to-good sensitivity and good specificity for the diagnosis of NAFLD in 23-year-old males and females across all ranges of nutritional status when compared with MRS. An area under curve of 86% indicates that this method has good diagnostic performance in the prediction of liver fat infiltration >5% in this age group. Analytical validation also showed that ultrasound was well suited to discriminate individuals with a cardiometabolic profile of high risk. Several authors highlight the potential of ultrasound in clinical contexts, in spite of reckoning its limitations [9,10,14,27,28]. Combined with a scoring system, the ultrasound method might serve as an initial screen for fatty liver in those at higher risk of having the condition, for instance people with: overweight or obesity, insulin resistance, prediabetes or type-2 diabetes, and high levels of serum triglycerides [9]. Second, it may serve to track the progression of the disease as well as grade its severity [10]. Last, ultrasound may help target and monitor interventions aiming at reducing the degree of liver steatosis and/or the cardiometabolic complications related to NAFLD [9,28].

We used MRS as the gold standard to determine liver fat since this is an accurate method, and liver biopsies could not be performed for ethical reasons in this group of participants without alterations in liver function. A recent study conducted among adolescents in whom a liver biopsy was performed, showed that MRS has a sensitivity and specificity of 92% and 95%, respectively, for the diagnosis of NAFLD [29]. A meta-analysis by Bohte et al. published in 2010 showed similar results [14]. Likewise, after revision of 49 studies including *n* = 4720 participants, Hernaez et al. found that ultrasound was reliable and accurate in detection of moderate and severe NAFLD, compared to biopsy, with an area under the summary receiving operating characteristics curve of 0.93 (0.91–0.95) [9].

Bohte et al. also found that ultrasound has better diagnostic accuracy than computed tomography (CT) scans. Other studies evaluating the accuracy of CT yielded similar results. A meta-analysis comparing CT with liver biopsies in liver donors showed that CT had a sensitivity and specificity of 0.81 and 0.94, respectively, and that the accuracy of CT increased when fat infiltration was higher [30]. Similarly, CT had a lower concordance with liver biopsies than ultrasound [31]. A semi-quantitative CT method, comparing liver and spleen radiological attenuation, could increase the precision of the technique [32]. This method was used in severely obese patients (BMI > 40 kg/m^2^). CT liver-spleen index was strongly correlated (−0.8; *p* < 0.001) to liver triglycerides, confirming the utility of using CT scanning to non-invasively evaluate the extent of liver fat infiltration in very high BMI patients. Therefore, the evidence shows that CT is not superior to ultrasound for the diagnosis of NAFLD. Considering that the later technique is far less expensive and avoids exposure to radiation, it should be preferred.

Ultrasound has the disadvantage of being operator dependent, since its results may vary according to the amount of gel used, the pressure exerted or the position of the probe [33]. The Hamaguchi score was designed to provide a numerical value for subjective parameters such as liver brightness or diaphragmatic attenuation. The two observers who determined the score in the present study had a moderate degree of concordance, providing evidence that this ultrasound method to determine liver fat overcomes interobserver variability. However, the concordance with MRS increased, notably when an agreement was reached between the raters. Therefore, having more than one observer may improve the accuracy of ultrasound. A recent report about liver fat assessment by ultrasound also showed a substantial level of concordance between observers when a similar semi-quantitative scoring method was used [34]. However, since the concordance between observers is not perfect, a good practice, especially for research purposes, is to have more than one assessment of images to reach an agreement when the scores are discordant.

When comparing participants with and without NAFLD, the former had higher BMI, serum lipids and insulin. The differences were more marked among men than women. This is in line with evidence describing a sexual dimorphism in the cardiometabolic profile, with males, generally showing less beneficial profiles [35].

### 4.2. Implications for Practice

Overweight and obesity are associated with a substantial risk of NAFLD in both older and younger age populations. The greater the severity of excess weight, the higher the risk of liver dysfunction, particularly among males. Clinicians should carefully examine patients who are overweight or obese to identify NAFLD early. Although elevated alanine transaminase has been proposed as a surrogate of NAFLD [36,37], in youths histologic studies and imaging procedures show that fatty liver is present in individuals with obesity whether the liver enzymes are elevated or not, suggesting that alanine transaminase elevation occurs at more advanced stages of NAFLD or in patients with extreme obesity [38,39,40]. Thus, abdominal ultrasound might be especially useful for screening of NAFLD in mild stages of the disease or in patients with obesity but still normal alanine transaminase levels. In our sample, alanine transaminase was slightly above normal (58.3 IU/L) only in males with NAFLD.

Likewise, significant risk factors for NAFLD are obesity, insulin resistance and the cardiometabolic risk factors that define the Metabolic Syndrome. The prevalence of these conditions has risen dramatically over the past years in both industrialized and non-industrialized countries, even among young adults. In Chile, 40% of 20–29 years-old have obesity, 14% have Metabolic Syndrome, one in three has low high-density lipoprotein cholesterol, and one in five has hypertriglyceridemia [4]. The number of young adults exposed to NAFLD is growing and, thus, more affordable and accessible methods for NAFLD screening in large groups are needed. This semi-quantitative ultrasound method to determine liver fat infiltration could serve that purpose which might help to discriminate individuals with mild NAFLD, for whom lifestyle interventions may have the potential to improve liver function. Moreover, screening of NAFLD using ultrasound could be done for preventive purposes in subjects with overweight.

### 4.3. Study Limitations

A major limitation of this study was the use of a relatively small sample (*n* = 60) to conduct the clinical validity assessment. Yet the number of participants was sufficient to address our main aim. Similar studies used similar or smaller sample sizes [15,41], and the analytical assessment was conducted to tackle this weakness, though the sample had less obesity, insulin resistance and Metabolic Syndrome. A larger sample might further clarify the predictive role of alanine aminotransferase in NAFLD among males. A further limitation was the use of a single ultrasound instrument, which does not allow assessing the variability that would be introduced by using a different ultrasound machine. The restricted age group might also limit the validity of our findings. However, the prevalence of NAFLD has substantially risen in young adults, a group where the disease often goes unrecognized and, if untreated, can progress eventually to steatohepatitis or cirrhosis before the age of 40 [6]. Lastly, due to the cross-sectional nature of our study, we were not able to assess the progression of NAFLD and confirm its relationship with biochemical and anthropometric markers over time.

## 5. Conclusions

Abdominal ultrasound, combined with a semi-quantitative score such as that proposed by Hamaguchi et al., is a reliable method to determine liver fat infiltration in young adults of any nutritional status. Its use should be encouraged in both clinical and epidemiological settings, and whenever MRS is unavailable.

## Figures and Tables

**Table 1:** Descriptive statistic of participants in the sample.

Variable	Participants Enrolled in the Clinical Validation (*n* = 60)		Participants Enrolled in the Analytical Validation (*n* = 555)		*p*
	Median/%	(IQR)/*n*	Median/%	(IQR)/*n*	
Age (years)	22.6	(22.3–22.7)	22.7	(22.3–22.9)	NS
Sex (male)	50%	30	51%	283	NS
Body-Mass Index	27.6	(23.3–32.5)	25.6	(22.5–29.6)	0.031
Waist circumference (cm)	82.9	(74.2–94.2)	80.7	(73.2–89.3)	NS
Systolic Blood Pressure (mm Hg)	116	(105–124)	110	(103–119)	0.038
Diastolic Blood Pressure (mm Hg)	70	(64–79)	70	(64–74)	NS
Fasting glucose (mg/dL)	90.7	(86.4–94.0)	88.0	(83.6–93.3)	0.032
Fasting insulin (uUI/ dL)	14.2	(9.2–20)	11.4	(7.7–16.1)	0.021
HOMA-IR	3.12	(2.0–4.5)	2.46	(1.6–3.6)	0.012
High-density lipoprotein cholesterol (mg/dL)	40.6	(32.2–48.8)	42.4	(34–51.8)	NS
Triglycerides (mg/dL)	86.1	(56.8–131.8)	84.4	(61.8–117.1)	NS
High-sensitivity C-reactive protein (mg/L)	1.57	(1.6–2.8)	1.34	(1.0–3.0)	NS
Alanine transaminase (IU/L)	37.2	(27.7–45.6)	33.7	(26.2–41.1)	<0.001
Aspartate transaminase (IU/L)	32.7	(26.1–46.3)	27.9	(21.0–37.2)	0.022
Hepatic fatty infiltration (%)	4.0	(2.0–12.5)	d.n.a	d.n.a	d.n.a
Hepatic fatty infiltration ≥5%	45%	27	d.n.a	d.n.a	d.n.a
Obesity (BMI ≥30)	36.6%	22	25.1%	139	0.027
Metabolic Syndrome (%)	28.3%	17	12.6%	70	0.022
Insulin Resistance (%)	61.2%	38	45.1%	250	0.014

Values are Median (interquartile range) and percentage/*n*. Metabolic Syndrome diagnosed with the International Diabetes Federation/American Heart Association/National Heart, Lung and Blood Institute joint standard. Insulin resistance diagnosed with HOMA-IR values ≥ 2.6. d.n.a: does not apply.

**Table 2:** Summary indices of the ultrasound performance for detection of non-alcoholic fatty liver disease (NAFLD) at a cut-off point ≥ 4.

	Overall	Males	Females
Sensitivity (%)	77.8	75.0	82.0
Specificity (%)	85.0	85.7	84.2
Correctly Classified (%)	81.7	80.0	83.3
Positive Predicted Value	81.0	85.7	75.0
Negative Predicted Value	82.4	75.0	90.0
False Positive Fraction	15.2	14.3	15.8
False Negative Fraction	22.0	25.0	18.2
Positive likelihood ratio	5.1	5.3	5.2
Negative likelihood ratio	0.3	0.3	0.2
Pre-test probability (prevalence)	45.0	53.3	36.7
Post-test probability (test positive)	80.8	85.7	75.0
Post-test probability (test negative)	17.6	25.0	11.1
Area Under Curve (%)	86.0	85.0	90.0
Cohen’s Kappa	0.63	0.61	0.65
Number of observations	60	30	30

**Table 3:** Anthropometric features, blood pressure and blood parameters in participants with and without NAFLD, according to Ultrasound (Hamaguchi score ≥ 4) expressed as median (interquartile range).

Variable	Overall sample (*n* = 60)		Females (*n* = 30)		Males (*n* = 30)	
	NAFLD (−) (*n* = 34)	NAFLD (+) (*n* = 26)	NAFLD (−) (*n* = 18)	NAFLD (+) (*n* = 12)	NAFLD (−) (*n* = 16)	NAFLD (+) (*n* = 14)
Body-Mass Index (kg/m^2^)	24.5	30.8 **	24.9	29.5 ‡	24	31.6 **
	(22.2–28.2)	(26.7–34.5)	(22.6–28.2)	(24.6–35.5)	(22–28)	(27.9–33.9)
Waist circumference (cm)	77.7	93.6 **	75.6	85.8 ‡	79.0	96.6 **
	(73.4–85.0)	(83.9–102.4)	(71.4–85.0)	(75.0–99.4)	(74.2–85.5)	(90.7–102.4)
Systolic Blood Pressure (mm Hg)	108.4	120.9 **	109.4	114.4	106.9	124.5 **
	(105–119)	(110–130)	(104.3–117)	(103–127)	(105–120.7)	(120–130)
Diastolic Blood Pressure (mm Hg)	68.4	78.5 **	65.4	66.7	69.3	80 **
	(63.3–70)	(68.3–80.7)	(64–70)	(61.2–82.2)	(60.8–76.3)	(78.3–80.7)
Blood glucose (mg/dL)	90.7	91	89.3	88.2	91.5	92.9
	(83.5–93.3)	(88–94.4)	(83.4–92.8)	(82.9–93)	(86.7–94.6)	(90–96.4)
Fasting insulin (uUI/dL)	12.1	19.8 **	13.4	17.5	11.3	21.8 **
	(8.8–16.3)	(11–38.6)	(10.1–18)	(10–45.5)	(8.6–14.2)	(13.1–38.6)
HOMA-IR (arbitrary units)	2.7	4.5 **	2.9	4	2.6	4.8 **
	(2–3.5)	(2.1–7.3)	(1.9–3.8)	(2.1–6.4)	(2–3.1)	(3.1–8.4)
Total Cholesterol (mg/dL)	158	165	164	166	149	166
	(130–184)	(138–196)	(130–187)	(138–202)	(127–176)	(138–191)
High- density lipoprotein cholesterol (mg/dL)	42.5	39.0	46.1	43.5	38.3	35.9
	(32.4–49.8)	(32.1–44.5)	(34.8–51)	(36–47.9)	(30.2–45.5)	(29.3–39.5)
Triglycerides (mg/dL)	78.5	105.5 ‡	86.7	85.6	64	135.3 ‡
	(60.2–105.2)	(56.3–182.6)	(65.9–105.2)	(51.3–129.7)	(56.5–109.6)	(66.4–196.8)
High-sensitivity C-reactive protein (mg/L) (*n* = 57)	1.57	1.70	1.87	2.02	1.29	1.61
	(1.1–2.7)	(1.2–3.3)	(1.2–2.4)	(1.2–3.3)	(1.1–2.8)	(1.2–6.0)
Adiponectin (µg/mL)	6.54	4.49	7.47	7.05	9.27	3.71
	(2.6–10.5)	(3.2–8.8)	(3.8 -8.8)	(2.6–8.69)	(2.6–9.27)	(1.7–5.5)
Alanine transaminase (IU/L)	29.5	38.0 **	31.3	32.7	28.4	58.3 **
	(25–36.5)	(31.5–66)	(26.1–36.5)	(24.9–38)	(23.9–38.3)	(32.9–88.8)
Aspartate transaminase (IU/L)	37.2	38.5	37.2	37.2	39.3	45.4
	(27.5–43.5)	(30.9–50.5)	(31.3–42.6)	(31.6–40.1)	(27–46.5)	(27.7–61.7)

Wilcoxson rank-sum test comparing participants with (+) and without (−) NAFLD. ** Significant at α of 0.01. ‡ Trend towards significance. Participants with high-sensitivity C-reactive protein values >9 mg/L were excluded from the analysis (*n* = 3).

**Table 4:** Cardiometabolic profile at 23y in male and female SLS participants with and without non-alcoholic fatty liver disease (NAFLD), according to abdominal ultrasound (NAFLD (+) or Hamaguchi score ≥ 4) (*n* = 555).

Variable	Males (*n* = 283)			Females (*n* = 272)		
	NAFLD (+)	NAFLD (−)	Effect Size ^†^	NAFLD (+)	NAFLD (−)	Effect Size ^†^
	(*n* = 57)	(*n* = 226)		(*n* = 75)	(*n* = 197)	
Waist circumference (cm)	94.1 ***	82.3	1.19	85.3 ***	77.4	0.86
	(11.6)	(8.8)		(9.9)	(8.9)	
Systolic Blood Pressure (mm Hg)	120.3 ***	113.5	0.70	110.8 *	107.8	0.32
	(9.9)	(9.7)		(9.5)	(9.3)	
Diastolic Blood Pressure (mm Hg)	75.1 ***	70.5	0.74	69.1 *	65.8	0.55
	(6.3)	(6.1)		(6.0)	(5.7)	
Fasting Glucose (mg/dL)	90.7 *	88.5	0.35	89.6 **	86.8	0.42
	(6.4)	(6.2)		(6.9)	(6.7)	
Fasting Insulin (uUI/dL)	14.8 ***	9.7	0.35 ^‡^	15.2 ***	11.7	0.33 ^‡^
	[12.9]	[6.5]		[12.8]	[7.8]	
HOMA-IR (arbitrary units)	3.4 ***	2.1	0.34 ^‡^	3.6 ***	2.5	0.31 ^‡^
	[3.2]	[1.5]		[3.4]	[1.7]	
High-density lipoprotein cholesterol (mg/dL)	38.7	41.3	d.n.a	44.5	47.8	d.n.a
	(11.9)	(12.3)		(12.9)	(12.1)	
Triglycerides (mg/dL)	92.1 *	82.1	0.28 ^‡^	108.4 *	86.1	0.26 ^‡^
	[59.8]	[53.3]		[74.4]	[51.6]	
high-sensitivity C-reactive protein (mg/L) (*n* = 480)	2.75 *	1.99	0.35	2.85	2.33	d.n.a
	(1.7)	(1.4)		(2.4)	(1.8)	
Adiponectin (µg/mL)	4.93 *	6.03	0.32	6.50 *	8.10	0.66
	(2.1)	(2.5)		(2.7)	(2.3)	

Values are Mean (standard deviations) and Median [interquartile range]. Student’s *t* test for comparison of mean values and Wilcoxson rank-sum test for comparison of median values. *** *p* value < 0.001, ** *p* value < 0.01, * *p* value < 0.05. † Cohen’s *d* statistic, except as indicated. ‡ Cliff’s δ statistic for non-normal distributions. Values of *d* of 0.20, 0.50 and 0.80 denote small, medium and large differences. Values of *δ* are considered small around 0.15, medium around 0.33, and large around 0.47. Participants with high-sensitivity C-reactive protein values >9.0 were excluded from the analysis *n* = 75).
